# Validity of the ACS NSQIP surgical risk calculator as a tool to predict postoperative outcomes in subacute orthopedic trauma diagnoses

**DOI:** 10.1016/j.heliyon.2024.e25796

**Published:** 2024-02-03

**Authors:** Charlotte L.E. Laane, Esther M.M. Van Lieshout, Roos A.M. Van Heeswijk, Amber I. De Jong, Michael H.J. Verhofstad, Mathieu M.E. Wijffels

**Affiliations:** Trauma Research Unit Dept. of Surgery, Erasmus MC, University Medical Center Rotterdam, Rotterdam, the Netherlands

**Keywords:** ACS NSQIP surgical risk calculator, Quality improvement, Trauma surgery, Urgency classification

## Abstract

**Purpose:**

This retrospective study aimed to validate the ACS NSQIP Surgical Risk Calculator (SCR) to predict 30-day postoperative outcomes in patients with one of the following subacute orthopedic trauma diagnoses; multiple rib fractures, pelvic ring/acetabular fracture, or unilateral femoral fracture.

**Methods:**

Data of patients with these diagnoses treated between January 1, 2015 and September 19, 2020 were extracted from the patients’ medical files. Diagnostic performance, discrimination, calibration, and accuracy of the ACS NSQIP SRC to predict specific outcomes developing within 30 days after surgery was determined.

**Results:**

The total cohort of the three diagnoses consisted of 435 patients. ACS NSQIP SRC underestimated the risk for serious complications, especially in patients with multiple rib fractures (8.3% predicted vs 17.2% observed) or pelvic ring/acetabular fracture (6.1% vs 19.8%). Underestimation was more pronounced for the composite outcome ‘any complication’. Sensitivity ranged from 16.7% to 100% and specificity from 41.1% to 97.1%. Specificity exceeded sensitivity for pelvic ring/acetabular and femoral fractures. Discrimination was good for predicting death (femoral fracture), fair for readmission (femoral fracture), serious complication (multiple rib fractures), and any complication (multiple rib fractures), but poor in all other outcomes and diagnoses. Calibration and accuracy were adequate for all three diagnoses (p-value for Hosmer-Lemeshow test >0.05 and Brier scores <0.25).

**Conclusion:**

Performance of the ACS NSQIP SRC in the studied cohort was variable for all three diagnoses. Although it underestimated the risk of most outcomes, calibration and accuracy seemed generally adequate. For most outcomes, adequate diagnostic performance and discrimination could not be confirmed.

## Highlights

• Performance of the ACS NSQIP SRC was variable for all three fracture types studied..
• The ACS NSQIP SRC underestimated the risk of most outcomes.
• Calibration and accuracy of the ACS NSQIP SRC were generally accurate.

## 1 Introduction

Patients with acute surgical conditions can have a disruptive effect on the elective surgical program. Especially for subacute diagnosis, discussing the order of the surgery program is challenging. This is partly due to the fact that literature on outcomes related to timing of surgery for patients with subacute diagnoses is inconclusive. Classification of urgency is critical, especially with limited treatment capacity [[1], [2], [3], [4], [5], [6], [7], [8], [9], [10], [11]].

Current classification systems merely use diagnosis-specific factors to define urgency [1,[12], [13], [14]]. Patient-related factors are hardly taken into account. In relation to subacute trauma diagnoses, it can be speculated that patients with a higher risk for postoperative complications might benefit from earlier surgery. In order to improve surgical care for vulnerable patients with a subacute orthopedic trauma diagnosis, a tool with consideration of patient-related risk factors should be used to triage patients. Accurately recognizing patients at a higher risk of postoperative complications is vital for health care management.

The American College of Surgeons developed the National Surgery Quality Improvement Program Surgical Risk Calculator (ACS NSQIP SRC) in order to predict postoperative events [15,16]. The SRC predicts postoperative outcomes accurately for pooled surgical diagnoses from the surgical subspecialties and for patients with colon surgery [16,17]. However, except for an accurate prediction of complications in elderly patients with a hip fracture, the validity of the ACS NSQIP SRC in specific subacute orthopedic trauma diagnoses requires investigation [18]. This includes commonly treated subacute conditions such as multiple rib fractures, pelvic ring/acetabular fractures, and femoral fractures. Subacute diagnoses are defined as critical conditions that are preferably operated on within 24 hours, present in patients who are otherwise stable.

The aim of this study was to determine the diagnostic performance, discrimination, calibration, and accuracy of the ACS NSQIP SRC to predict 30-day postoperative outcomes in patients with a subacute diagnosis of multiple rib fractures, pelvic ring/acetabular fracture, or unilateral femoral fracture.

## 2 Methods

This retrospective cohort study was performed in an academic hospital. Patients were identified from the patient's medical files, using the registered local surgical codes for the diagnoses multiple (*i.e.*, three or more) rib fractures, pelvic ring/acetabular fracture, or unilateral femoral fracture.

Patients aged 18 years or older who underwent urgent (definitive) surgical treatment for any of the three predefined diagnoses after trauma between January 1, 2015 and September 19, 2020 were included. All procedures were designated as emergency cases that lacked other potential appropriate treatment options. Patients with insufficient data to compute the ACS NSQIP SRC score, patients who were pregnant during surgery, patients who required immediate surgery based on hemodynamic instability, and patients for whom less than 30 days of postoperative follow-up was available in their medical files were excluded. Preoperative comorbidities and postoperative events were collected from the patient's medical files. Postoperative events were restricted to events that were directly related (or highly likely directly related) to the diagnosis studied.

The study was exempted by the Medical Research Ethics Committee (ref.no. MEC-2020-0430), and consent was waived. The study has been performed in accordance with the ethical standards laid down in the 1964 Declaration of Helsinki and its later amendment.

## 3 ACS NSQIP SRC

**Fig. 1:**
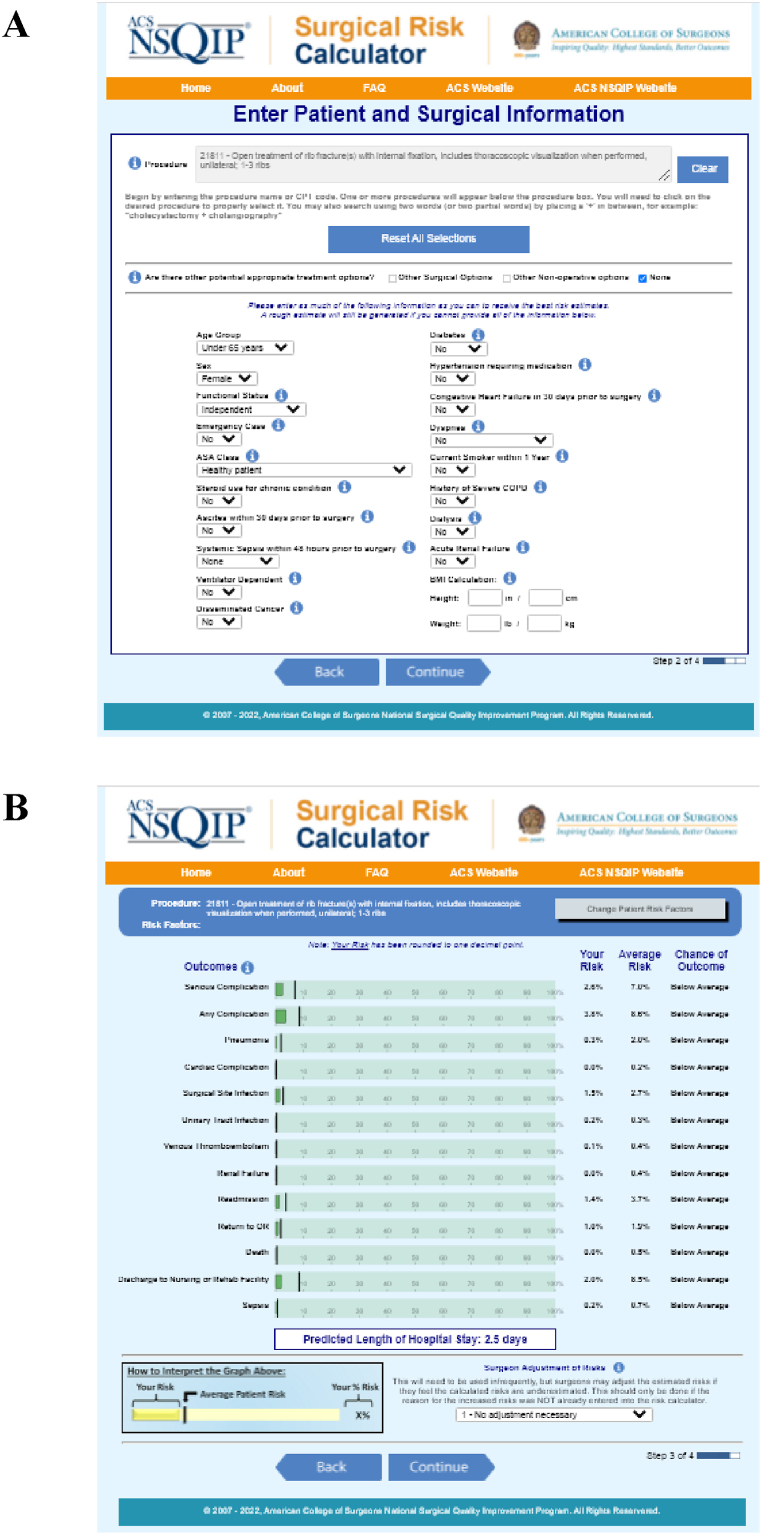
Example of the ACS NSQIP SRC website, showing the A) patient and surgical information and B) resulting risks of postoperative outcomes.

The ACS NSQIP SRC predicts postoperative (adverse) events based on Current Procedural Terminology (CPT®) codes and 20 preoperative factors, including demographic information and comorbidities (Fig. 1A). The SRC is based on the ACS NSQIP patient database, which includes more than 6 million patients with various diagnoses in 719 hospitals worldwide, evaluated in 2019 [19]. The calculator scores patient-specific and average predicted risks of 13 postoperative (adverse) events within 30 days after surgery or hospital length of stay (HLOS; Fig. 1B) [20]. The definition for each comorbidity and outcome is given on the ACS NSQIP SRC website as part of the calculator. The outcomes are combined into two composite outcome measures, being serious complication or any complication [19,20]. In 14% of patients, ASA class was determined using alternative information from medical files. An overview of the CPT® codes with description used in this study is shown in Table 1.

**Table 1:** Frequency of Current Procedural Terminology (CPT) codes per diagnosis in the study population.

Diagnosis	CPT		Frequency
	Code	Description	
Multiple rib fractures (n = 58)	21811	Open treatment of rib fracture(s) with internal fixation, includes thoracoscopic visualization when performed, unilateral; 1–3 ribs	58 (100.0%)
Pelvic ring/acetabular fracture (n = 116)	27215	Open treatment of iliac spine(s), tuberosity avulsion, or iliac wing fracture(s), unilateral, for pelvic bone fracture patterns that do not disrupt the pelvic ring, includes internal fixation, when performed	4 (3.4%)
	27217	Open treatment of anterior pelvic bone fracture and/or dislocation for fracture patterns that disrupt the pelvic ring, unilateral, includes internal fixation, when performed (includes pubic symphysis and/or ipsilateral superior/inferior rami)	35 (30.2%)
	27218	Open treatment of posterior pelvic bone fracture and/or dislocation, for fracture patterns that disrupt the pelvic ring, unilateral, includes internal fixation, when performed (includes ipsilateral ilium, sacroiliac joint and/or sacrum)	2 (1.7%)
	27226	Open treatment of posterior or anterior acetabular wall fracture, with internal fixation	27 (23.3%)
	27227	Open treatment of acetabular fracture(s) involving anterior or posterior (one) column, or a fracture running transversely across the acetabulum, with internal fixation	29 (25.0%)
	27228	Open treatment of acetabular fracture(s) involving anterior and posterior (two) columns, includes T-fracture and both column fracture with complete articular detachment, or single column or transverse fracture with associated acetabular wall fracture, with internal fixation	11 (9.5%)
	27254	Open treatment of hip dislocation, traumatic, with acetabular wall and femoral head fracture, with or without internal or external fixation	1 (0.9%)
	27280	Arthrodesis, open, sacroiliac joint, including obtaining bone graft, including instrumentation, when performed	7 (6.0%)
Femoral fracture (n = 261)	27236	Open treatment of femoral fracture, proximal end, neck, internal fixation or prosthetic replacement	91 (34.9%)
	27244	Treatment of intertrochanteric, peritrochanteric, or subtrochanteric femoral fracture, with plate/screw type implant, with or without cerclage	44 (16.9%)
	27245	Treatment of intertrochanteric, peritrochanteric, or subtrochanteric femoral fracture, with intramedullary implant, with or without interlocking screws or cerclage	44 (16.9%)
	27269	Open treatment of femoral fracture, proximal end, head, includes internal fixation, when performed	1 (0.4%)
	27506	Open treatment of femoral shaft fracture, with or without external fixation, with insertion of intramedullary implant, with or without cerclage or locking screws	55 (21.1%)
	27507	Open treatment of femoral shaft fracture with plate/screws, with or without cerclage	12 (4.6%)
	27511	Open treatment of femoral supracondylar or transcondylar fracture without intercondylar extension, includes internal fixation when performed	8 (3.1%)
	27513	Open treatment of femoral supracondylar or transcondylar fracture with intercondylar extension, includes internal fixation, when performed	6 (2.3%)

Data are shown as n (%).

## 4 Outcome measures

The primary outcome measure of this study was the predictive value of the ACS NSQIP SRC score for the different (adverse) events within 30 days or HLOS, defined by diagnostic performance, discrimination, calibration, and accuracy. All complications within 30 days were collected, including those that occurred during this timeframe but were documented after this period. The secondary outcome measure was the time to surgery and its correlation with the ACS NSQIP SRC score.

## 5 Statistical analysis

Data were analyzed using the Statistical Package for the Social Sciences (SPSS) version 25 (SPSS, Chicago, Ill., USA). Receiver operating characteristic (ROC) analysis and calculation of sensitivity and specificity were performed using MedCalc Statistical Software version 18.2.1 (MedCalc Software bvba, Ostend, Belgium; http://www.medcalc.org; 2018). Normality of continuous variables was tested with the Shapiro-Wilk test. A p-value lower than 0.05 was considered statistically significant. Missing data were not imputed.

First, demographics and patient characteristics were summarized using descriptive statistics. Data are shown as median with quartiles for continuous variables and numbers with percentage for categorical variables.

Next, the correlation between the ACS NSQIP SRC predicted risk and the time to surgery was determined using the Spearman rank correlation test. Spearman's Rho is shown separately for patients who did or did not develop the (adverse) events studied.

Finally, the performance of the ACS NSQIP SRC in predicting the 30-day risk of postoperative outcome was evaluated. The SRC predicted risk for (adverse) events (serious, any, and specific (adverse) events as mentioned above) was compared against the actual observed value (i.e., whether the complication occurred or did not occur). The diagnostic performance, discrimination, calibration, and accuracy of the SRC were evaluated for the three studied diagnoses analogous to procedures used in the original validation of the SRC [16]. For diagnostic performance, sensitivity and specificity were computed. For this analysis, the predicted risk was categorized as 1) “above average risk” versus 2) “average risk or below average risk”.

Discrimination was evaluated by the area under the receiver operating characteristic curve (AUC) or c-statistic. The curve plots the false-positive rate (1-specificity) against the true-positive rate (sensitivity) for all possible predicted risk score cutoff points. The SRC predicted risk per patient was entered as continuous variable. An AUC of 0.5 indicates no discrimination above chance and an AUC of 1.0 indicates perfect discrimination. Generally, an AUC = 0.9–1.0 represents excellent, AUC = 0.8–0.9 good, AUC = 0.7–0.8 fair, and AUC = 0.6–0.7 poor discriminative ability. Discrimination is assumed to be useful if AUC ≥0.75 [21].

Calibration was assessed performing the Hosmer-Lemeshow goodness-of-fit test. The Hosmer-Lemeshow (HL) statistic evaluates differences in the probability of observed and predicted events across deciles of increasing predicted risk. The SRC predicted risk was entered as continuous variable. The null hypothesis that the SRC model is well-calibrated is rejected at a p-value of <0.05 [22].

The accuracy of the SRC was assessed using the Brier score, which reflects the deviation between the predicted and observed (adverse) events. The Brier score is computed as the mean squared differences between the predicted risk (continuous variable) and the actual outcome. Presence or absence of an event was scored as 1 or 0, respectively. A risk prediction model that perfectly predicts the outcomes of all individuals has a Brier score of 0. A Brier score of 1 indicates that the model did not predict the outcome. A score <0.01 indicates predictive precision >90% [15,23,24]. A risk prediction model with a Brier score of 0.25 or higher is considered non-informative [22].

## 6 Results

**Fig. 2:**
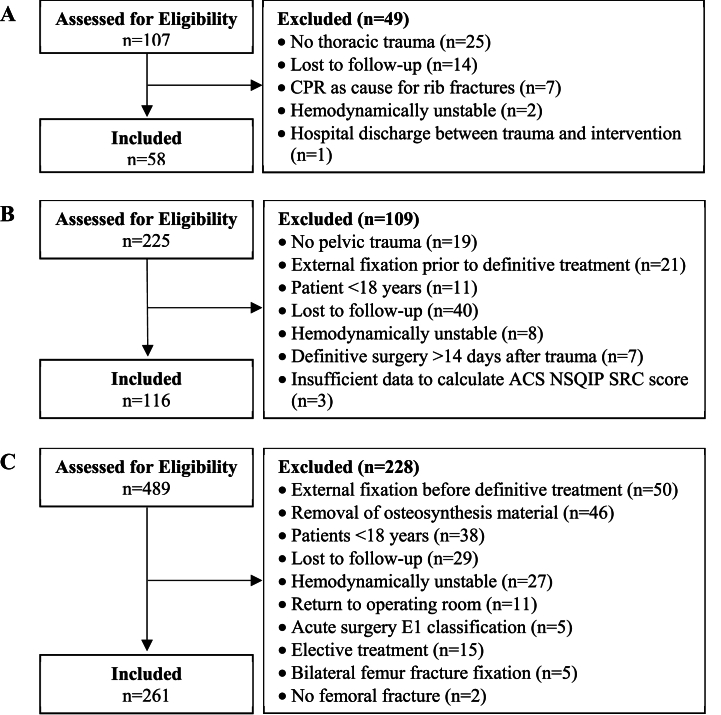
Flow chart for A) multiple rib fractures, B) pelvic ring/acetabular fracture, or C) femoral fracture.

**Fig. 3:**
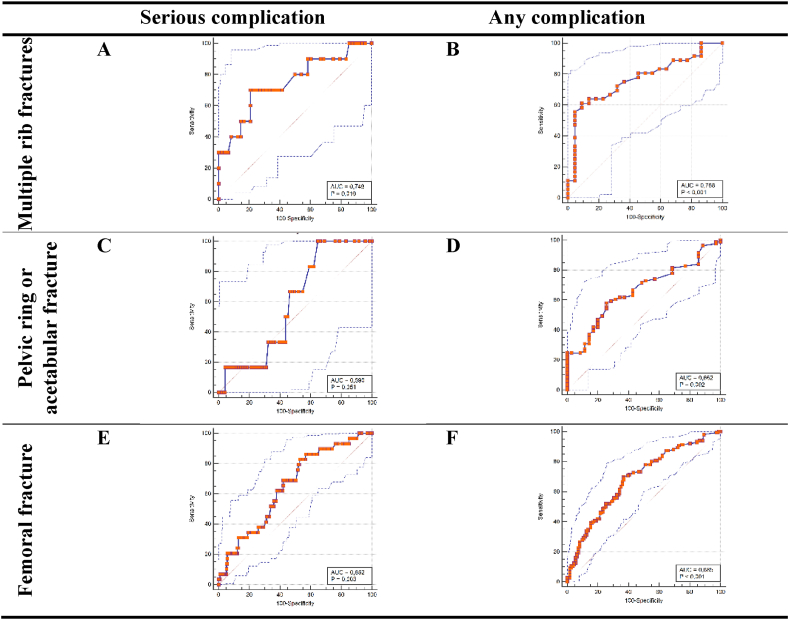
Receiver-operating characteristic curves for serious or any postoperative complication according to the ACS NSQIP SRC for A and B) multiple rib fractures, C and D) pelvic ring/acetabular fracture, or E and F) femoral fracture.

**Fig. 4:**
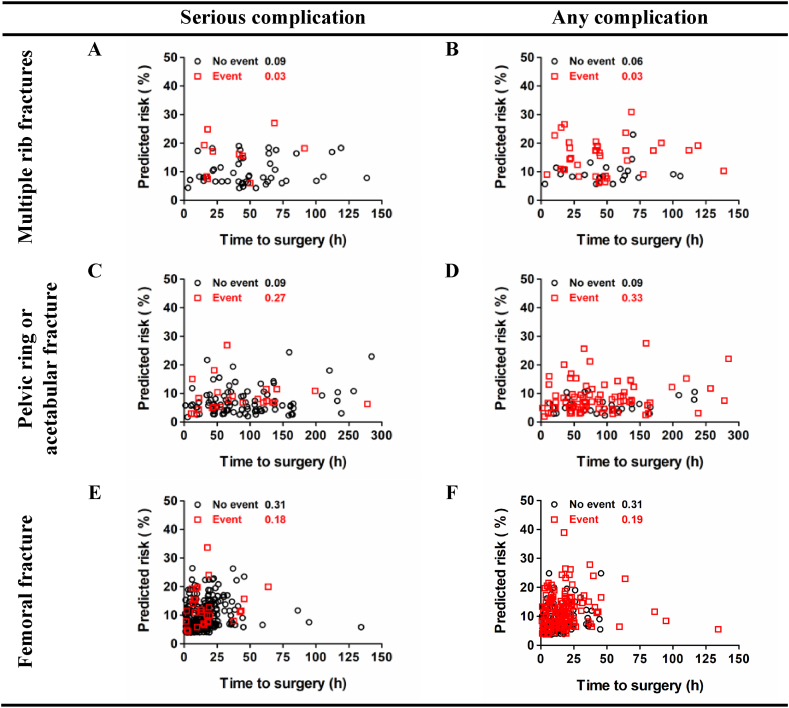
Correlation between ACS NSQIP SRC predicted risk for serious or any complication and time to surgery for A and B) multiple rib fractures, C and D) pelvic ring/acetabular fracture, or E and F) femoral fracture.

The overall number of patients for all three diagnoses included in the analysis was 435. Below, more detailed results are given, stratified for the three studied diagnoses. Fig. 2 shows the study flow chart per diagnosis. Table 2 provides an overview of the patient characteristics and preoperative risk factors per diagnosis. Online Supplemental Table S1 shows the observed rates and risks predicted by the ACS NSQIP SRC for all 13 outcomes per diagnosis, in the order that the calculator presents those outcomes. In addition, it shows the diagnostic performance, discrimination, calibration, and accuracy of the ACS NSQIP SRC for predicting these postoperative (adverse) events. The results section below focuses on the three main (adverse) events, which are presented in Table 3. Fig. 3, Fig. 4 show the ROC curves and correlations of ACS NSQIP SRC predicted risk with time to surgery for the serious and any complication of the three diagnoses studied, respectively.

**Table 2:** Preoperative risk factors per diagnosis.

Characteristic	Multiple rib fractures (n = 58)	Pelvic ring/acetabular fracture (n = 116)	Femoral fracture (n = 261)
Baseline characteristics			
Age (years)	60 (50–69)	43 (30–57)	64 (43–76)
Age			
<65 years	39 (67.2%)	99 (85.3%)	132 (50.6%)
65–74 years	10 (17.2%)	14 12.1%)	52 (19.9%)
67–84 years	9 (15.5%)	3 (2.6%)	51 (19.5%)
≥ 85 years	0 (%)	0 (0.0%)	26 (10.0%)
Female sex	14 (24.1%)	27 (23.3%)	104 (39.8%)
Functional status			
Independent	58 (100.0%)	115 (99.1%)	220 (84.3%)
Partially dependent	0 (0.0%)	1 (0.9%)	35 (13.4%)
Totally dependent	0 (0.0%)	0 (0.0%)	6 (2.3%)
ASA class			
ASA 1	6 (10.3%)	44 (37.9%)	48 (18.4%)
ASA 2	22 (37.9%)	48 (41.4%)	101 (38.7%)
ASA 3	16 (27.6%)	18 (15.5%)	97 (37.2%)
ASA 4	14 (24.1%)	6 (5.2%)	14 (5.4%)
ASA 5	0 (0.0%)	0 (0.0%)	1 (0.4%)
BMI (kg/m^2^)^a^	25.7 (23.1–29.2)	24.7 (22.7–27.6)	23.9 (21.5–26.8)
***Comorbidities***			
Steroid use for chronic condition	4 (6.9%)	3 (2.6%)	26 (10.0%)
Ascites <30 days preoperatively	0 (0.0%)	0 (0.0%)	1 (0.4%)
Systemic sepsis <48 h preoperatively			
SIRS	1 (1.7%)	0 (0.0%)	0 (0.0%)
Sepsis	0 (0.0%)	1 (0.9%)	1 (0.4%)
Septic shock	0 (0.0%)	0 (0.0%)	0 (0.0%)
Ventilator dependent	20 (34.5%)	19 (16.4%)	13 (5.0%)
Disseminated cancer	0 (0.0%)	0 (0.0%)	29 (11.1%)
Diabetes requiring medication			
Oral	3 (5.2%)	6 (5.2%)	29 (11.1%)
Insulin	4 (6.9%)	1 (0.9%)	17 (6.5%)
Hypertension requiring medication	16 (27.6%)	15 (12.9%)	66 (25.3%)
Congestive heart failure <30 days preoperatively	2 (3.4%)	1 (0.9%)	7 (2.7%)
Dyspnea			
With moderate exertion	1 (1.7%)	1 (0.9%)	15 (5.7%)
At rest	0 (0.0%)	0 (0.0%)	5 (1.9%)
Current smoker <1 year	12 (20.7%)	32 (27.6%)	66 (25.3%)
History of severe COPD	2 (3.4%)	1 (0.9%)	17 (6.5%)
Dialysis	1 (1.7%)	1 (0.9%)	3 (1.1%)
Acute renal failure	2 (3.4%)	3 (2.6%)	4 (1.5%)
***Miscellaneous***			
Time from diagnosis to surgery (h)	44.3 (22.4–64.6)	73.7 (45.2–124.1)	14.1 (5.9–19.8)
Duration of surgery (min)	116 (96–167)	210 (169–307)	129 (101–188)
ICU admission^b^	34 (58.6%)	25 (21.6%)	42 (16.1%)
ICU LOS (days)	9 (3–18)	5 (2–18)	(2–5)
HLOS (days)	18 (10–32)	13 (10–22)	8 (5–13)

Data are shown as median (P_25_–P_75_) or as n (%).

ASA, American Society of Anesthesiologists; BMI, body mass index; COPD, chronic obstructive pulmonary disease; HLOS, hospital length of stay; ICU, intensive care unit; LOS, length of stay; SIRS, systemic inflammatory response syndrome.

a Data are missing for 11 patients with a femoral fracture.

b Data are shown for patients admitted to the ICU.

**Table 3:** Observed rates and predicted risks of postoperative outcomes and diagnostic performance, discrimination, and calibration of the ACS NSQIP SRC.

Outcome	Risk of outcome		Diagnostic performance		Discrimination	Calibration	Accuracy
	Observed rate n (%)	Predicted risk (%; P_25_–P_75_)	Sensitivity (95% CI)	Specificity (95% CI)	AUC (95% CI; p-value)	HL-test Chi^2^ (p-value)	Brier score
*Multiple rib fractures (n = 58)*							
Serious complication	10 (17.2%)	**8.3 (6.6–16.4)**	80.0 (44.4–97.5)	52.1 (37.2–66.7)	0.75 (0.62–0.85; **0.010**)	4.161 (0.761)	0.132
Any complication	36 (62.1%)	**11.0 (8.4–17.5)**	66.7 (49.0–81.4)	72.7 (49.8–89.3)	0.77 (0.64–0.87; **<0.001**)	7.456 (0.488)	0.451
Readmission	4 (6.9%)	**4.8 (3.3–5.7)**	25.0 (0.6–80.6)	70.4 (56.4–82.0)	0.57 (0.43–0.70; 0.514)	6.142 (0.631)	0.065
Return to OR	2 (3.4%)	3.1 (2.7–4.2)	50.0 (1.3–98.7)	41.1 (28.1–55.0)	0.51 (0.37–0.64; 0.976)	8.280 (0.309)	0.033
Death	1 (1.7%)	**0.2 (0.1–1.4)**	100.0 (2.5–100.0)	52.6 (39.0–66.0)	N.D.	0.000 (N.D.)	0.015
*Pelvic ring/acetabular fracture (n = 116)*							
Serious complication	23 (19.8%)	**6.1 (4.3–9.4)**	30.4 (13.2–52.9)	76.3 (66.4–84.5)	0.64 (0.55–0.73; **0.021**)	12.149 (0.145)	0.171
Any complication	81 (69.8%)	**6.5 (4.5–9.5)**	30.9 (21.1–42.1)	97.1 (85.1–99.9)	0.66 (0.57–0.75; **0.002**)	9.637 (0.291)	0.588
Readmission	6 (5.2%)	**2.7 (1.9–3.9)**	16.7 (0.4–64.1)	90.9 (83.9–95.6)	0.69 (0.60–0.78; **0.008**)	9.279 (0.233)	0.049
Return to OR	19 (16.4%)	**2.6 (1.5–3.5)**	47.4 (24.4–71.1)	67.0 (56.7–76.2)	0.65 (0.56–0.74; **0.035**)	8.204 (0.414)	0.154
Death	1 (0.9%)	**0.1 (0.0–0.4)**	100.0 (2.5–100.0)	77.4 (68.7–84.7)	N.D.	0.142 (0.998)	0.008
*Femoral fracture (n = 261)*							
Serious complication	29 (11.1%)	8.7 (6.0–13.1)	37.9 (20.7–57.7)	69.4 (63.0–75.3)	0.65 (0.59–0.71; **0.003**)	5.783 (0.672)	0.096
Any complication	150 (57.5%)	**8.4 (5.8–13.3)**	40.0 (32.1–48.3)	82.9 (74.6–89.4)	0.69 (0.63–0.74; **<0.001**)	6.012 (0.646)	0.456
Readmission	9 (3.4%)	4.6 (2.7–7.7)	66.7 (29.9–92.5)	72.2 (66.3–77.7)	0.75 (0.69–0.80; **0.005**)	6.518 (0.589)	0.033
Return to OR	16 (6.1%)	**1.7 (1.2–2.1)**	50.0 (24.7–75.3)	61.6 (55.2–67.8)	0.56 (0.50–0.62; 0.393)	3.305 (0.855)	0.059
Death	13 (5.0%)	**0.8 (0.2–3.6)**	76.9 (46.2–95.0)	70.2 (64.0–75.8)	0.83 (0.78–0.88; **<0.001**)	11.285 (0.127)	0.043

### 6.1 Multiple rib fractures

In the group with multiple rib fractures, 58 out of 107 patients screened were included (Fig. 2A). They had a median age of 60 years (P_25_–P_75_ 50–69), and 14 (24.1%) patients were female (Table 2). The median time to surgery was 44.3 h (P_25_–P_75_ 22.4–64.6).

The observed complication rates were up to 5 times higher than the risks predicted by the ACS NSQIP SRC for the outcomes listed in Table 3. As a measure of diagnostic performance, sensitivity ranged from 25.0% for readmission to 100% for death (Table 3). Specificity was lower, and ranged from 41.1% for return to OR to 72.7% for any complication. The discriminatory ability was very poor for readmission and return to OR (AUC <0.6) and fair for serious complication and any complication (AUC 0.7–0.8; Table 3 and Fig. 3A and B). Calibration was adequate for the outcomes listed (HL > 0.05) (Table 3). The ACS NSQIP SRC showed adequate predictive precision for the outcomes mentioned (*i.e.*, Brier score <0.25), except for any complication (Table 3).

No statistically significant associations were found between predicted risk scores (for serious or any complication) and time to surgery (Fig. 4A and B).

### 6.2 Pelvic ring/acetabular fractures

In the group with a pelvic ring/acetabular fracture, 116 out of 225 patients screened were included (Fig. 2B). They had a median age of 43 years (P_25_–P_75_ 30–57), and 27 (23.3%) patients were female (Table 2). The median time to surgery was 73.7 h (P_25_–P_75_ 45.2–124.1).

The observed complication rates were higher than the risks predicted by the ACS NSQIP SRC for the outcomes listed in Table 3. The largest effect was seen for any complication (69.8% observed vs 6.5% predicted). Sensitivity ranged from 16.7% for readmission to 100% for death (Table 3). Specificity was higher, and ranged from 67.0% for return to OR to 97.1% for any complication. The AUC ranged from 0.64 to 0.69, which indicates poor discriminatory ability for the outcomes listed (Table 3 and Fig. 3C and D). The p-value of the HL test was consistently >0.05, indicating that the ACS NSQIP SRC has adequate calibration for the outcomes listed (Table 3). For the prediction of postoperative (adverse) events, the ACS NSQIP SRC showed adequate predictive precision (*i.e.*, Brier score <0.25), except for any complication (Table 3).

No statistically significant associations were found between predicted risk scores (for serious or any complication) and time to surgery (Fig. 4C and D).

### 6.3 Femoral fractures

In the group with a femoral fracture, 261 out of 489 patients screened were included (Fig. 2C). They had a median age of 64 years (P_25_–P_75_ 43–76), and 104 (39.8%) patients were female (Table 2). The median time to surgery was 14.1 h (P_25_–P_75_ 5.9–19.8).

The observed complication rates were higher than the risks predicted by the ACS NSQIP SRC for the outcomes listed in Table 3, except for readmission (3.4% observed versus 4.6% predicted). Sensitivity ranged from 37.9% for serious complication to 76.9 % for death (Table 3). Specificity was higher, except for death, and ranged from 61.6% for return to OR to 82.9% for any complication. The discriminatory ability was (very) poor for serious complication, any complication, and return to OR (AUC<0.7), fair for readmission (AUC 0.7–0.8), and good for death (AUC 0.8–0.9; Table 3 and Fig. 3E and F). Calibration was adequate for all outcomes listed (Table 3). The ACS NSQIP SRC showed adequate predictive precision for the outcomes mentioned (*i.e.*, Brier score <0.25), except for any complication (Table 3).

The predicted risk scores (for serious or any complication) were statistically significantly positively correlated with time to surgery (Fig. 4E and F).

## Discussion

The results of this study show that the predicted risk by the ACS NSQIP SRC is an underestimation of the observed rate of postoperative outcomes in all three diagnoses studied. Sensitivity and specificity varied highly across the outcomes and diagnoses, with sensitivity ranging from 16.7% to 100% and specificity ranging from 41.1% to 97.1%. The discriminatory ability was good for predicting death (femoral fracture) and fair for readmission (femoral fracture), serious complication (multiple rib fractures), and any complication (multiple rib fractures), but poor in all other outcomes and diagnoses. Calibration was adequate for all outcomes in all diagnoses. Finally, the accuracy was adequate for all outcomes studied, except for any complication (multiple rib fractures, pelvic ring/acetabular fracture, and femoral fracture). These results suggest that the ACS NSQIP SRC is not a suitable risk prediction tool for these three diagnoses in an academic hospital setting in The Netherlands.

A statistically significant positive association of the ACS NSQIP SRC predicted risk with time to surgery was found for patients with a femoral fracture (*i.e.*, patients with a lower ACS NSQIP SRC score were operated earlier). A possible explanation would be that patients with a lower complication risk have less comorbidities and would thus require a less comprehensive preoperative preparation. There is a paucity in literature on the performance of the ACS NSQIP SRC for the subacute diagnoses investigated in the current study.

It is unclear if the underestimation of the complication rates may, to some extent, be due to cross-cultural differences, or if it is attributable to a different case mix between the current cohort (*i.e.*, academic setting with a substantial proportion of polytrauma) and the population used for determining the weighting factors in the SRC (*i.e.*, general population with mostly monotrauma). Similar to the current data on femoral fractures, Wang et al. showed that the ACS NSQIP SRC underestimates the risk of postoperative outcomes, adequate calibration for all outcomes, and adequate accuracy for all outcomes except any complication [18]. Their results on discrimination differed from our results for serious complication and any complication (poor in our study versus fair for Wang et al.), readmission (fair versus poor), return to OR (poor versus good), and death (good versus excellent). It is unclear if this is attributable to differences in age (65 years or older, which comprises only 50% of our study) and the much larger sample size, or a potential difference in case mix between the two studies. To our knowledge, no research specifically focusing on using the ACS NSQIP SRC for multiple rib fractures and pelvic ring/acetabular fractures is available. However, in comparison with previous literature this cohort had a higher overall complication rate for multiple rib fractures of 62.1% versus 10.3%, and a higher pneumonia rate of 27.6% vs 16.7 and 17.1% [25,26]. This is similar for pelvic ring fractures and femoral fractures where literature reports 7.0–31.1% and 25.3–35.5% complication rates versus 69.8% and 57.5% in this cohort [[27], [28], [29]]. This further supports that the ACS NSQIP SRC is not a good tool to predict complications in an academic hospital in The Netherlands.

The results of the different performance measures indicate that implementation of the ACS NSQIP SRC in a subacute orthopedic trauma setting requires more extensive validation studies. In the current study, the ACS NSQIP SRC has not been used for timing of surgery, and the data generally show no significant association between the time to surgery and the predicted risk of postoperative outcomes. Literature shows that early surgical treatment results in lower risk of complications [2,4,8,11]. Whether or not implementing the ACS NSQIP SRC as a tool to optimize surgical timing will reduce the rates of postoperative outcomes (and consequently the difference between observed rates and predicted risks) requires further investigation.

As with most observational studies, this study also had some limitations, the most obvious one being the retrospective design. Excluding patients due to missing data for the ACS NSQIP SRC and due to loss to follow-up may have affected the true rates of postoperative outcomes. In addition, with the participating site being an academic hospital, the enrolled patient group likely does not fully represent the general population with any of the three diagnoses included. A substantial proportion has additional injuries, making it hard to link the complications to the studied diagnoses [30,31]. The ACS NSQIP SRC has added the “Surgeon adjustment of risk” variable, there was however no standardized method to incorporate this due to the retrospective nature of this study. In addition, there is a possibility that patients present their complications within 30 days at a different healthcare facility. In the event they sought medical attention elsewhere, this would have been registered during their subsequent follow-up appointment and included in the current analysis. Additional research is needed in order to draw conclusions on the validity of the ACS NSQIP SRC for the general population with these diagnoses. Another limitation is in the ACS NSQIP SRC itself. The calculator not only incorporates native complications, but also dependent sequelae, such as return to OR, discharge to nursing or rehabilitation facility, and death, which are not complications but consequences thereof. In addition, the use of composite outcome measures like ‘any complication’ any ‘serious complication’ has limitation, especially in the current population in which participants may have multiple injuries. Furthermore, with the relatively small sample size of multiple rib fractures and an associated low number of some of the individual outcomes, this study could be underpowered. Larger, preferably prospective, studies are required in order to draw a final conclusion on the validity of the ACS NSQIP SRC for predicting outcomes in this specific set of injuries. There is no available prior literature for these diagnoses to support external validity. Finally, literature reporting on the validity of the ACS NSQIP SRC for multiple rib fractures and pelvic ring/acetabular fractures is limited. Therefore, this study adds valuable information to literature. The strengths of this study include the limited amount of missing data and therefore the ability to compute the ACS NSQIP SRC predicted risk for outcomes in most patients. This results in an accurate representation of patient demographics within the academic hospital in this study for these diagnoses.

In conclusion, results of the current study show that performance of the ACS NSQIP Surgical Risk Calculator is variable for all three diagnoses studied in this cohort. Although it underestimates most postoperative outcomes in our patient population, calibration and accuracy seem generally adequate. For the majority of outcomes, adequate diagnostic performance and discrimination could not be confirmed. The results indicate that within this cohort in an academic hospital setting in The Netherlands, the ACS NSQIP SRC might not be the most appropriate tool for predicting risks related to these three diagnoses.

## Statement of informed consent

The local Medical Research Ethics Committee Erasmus MC exempted the study and waived consent (MEC-2020-0430).

## CRediT authorship contribution statement

**Charlotte L.E. Laane:** Writing – review & editing, Writing – original draft, Investigation, Conceptualization. **Esther M.M. Van Lieshout:** Writing – review & editing, Writing – original draft, Supervision, Project administration, Methodology, Formal analysis. **Roos A.M. Van Heeswijk:** Writing – review & editing, Data curation. **Amber I. De Jong:** Writing – review & editing, Data curation. **Michael H.J. Verhofstad:** Writing – review & editing, Supervision, Conceptualization. **Mathieu M.E. Wijffels:** Writing – review & editing, Writing – original draft, Supervision, Conceptualization.

## Declaration of competing interest

The authors declare that they have no known competing financial interests or personal relationships that could have appeared to influence the work reported in this paper.
