# Supplementary material for: Validity of the ACS NSQIP surgical risk calculator as a tool to predict postoperative outcomes in subacute orthopedic trauma diagnoses
Source: Heliyon. 2024 Feb 3;10(4):e25796. doi: 10.1016/j.heliyon.2024.e25796 (PMC10875421; doi:10.1016/j.heliyon.2024.e25796)
Supplement: Multimedia component 1 [file mmc1.docx]

**Supplemental Table S1: Observed rates and predicted risks of postoperative outcomes and diagnostic performance, discrimination, and calibration of the ACS NSQIP SRC; full overview of all outcomes included in the SRC**

|  | **Risk of outcome** | | **Diagnostic performance** | | **Discrimination** | **Calibration** | **Accuracy** |
| --- | --- | --- | --- | --- | --- | --- | --- |
| **Outcome** | **Observed rate**  **n (%)** | **Predicted risk**  **(%; P_25_-P_75_)** | **Sensitivity**  **(95% CI)** | **Specificity**  **(95% CI)** | **AUC**  **(95% CI; p-value)** | **HL-test**  **Chi^2^ (p-value)** | **Brier score** |
| ***Multiple rib fractures (n=58)*** |  |  |  |  |  |  |  |
| Serious complication | 10 (17.2%) | **8.3 (6.6-16.4)** | 80.0 (44.4-97.5) | 52.1 (37.2-66.7) | 0.75 (0.62-0.85; **0.010**) | 4.161 (0.761) | 0.132 |
| Any complication | 36 (62.1%) | **11.0 (8.4-17.5)** | 66.7 (49.0-81.4) | 72.7 (49.8-89.3) | 0.77 (0.64-0.87; **<0.001**) | 7.456 (0.488) | 0.451 |
| Pneumonia | 16 (27.6%) | **2.1 (1.3-5.4)** | 75.0 (47.6-92.7) | 52.4 (36.4-68.0) | 1.00 (0.94-1.00; **<0.001**) | 7.253 (0.403) | 0.250 |
| Cardiac complication | 1 (1.7%) | **0.3 (0.1-0.8)** | 100.0 (2.5-100.0) | 50.9 (37.3-64.4) | N.D. | 8.435 (0.208) | 0.017 |
| Surgical site infection | 2 (3.4%) | 3.3 (2.7-4.1) | 0.0 (0.0-84.2) | 78.6 (65.6-88.4) | 0.51 (0.37-0.64; 0.975) | 6.253 (0.511) | 0.033 |
| Urinary tract infection | 4 (6.9%) | **0.5 (0.3-0.6)** | 75.0 (19.4-99.4) | 46.3 (32.6-60.4) | 0.59 (0.45-0.71; 0.644) | 3.213 (0.667) | 0.068 |
| Venous Thromboembolism | 6 (10.3%) | **0.4 (0.3-0.7)** | 100.0 (54.1-100.0) | 69.2 (54.9-81.3) | 0.86 (0.74-0.94; **<0.001**) | 4.752 (0.576) | 0.102 |
| Renal failure ^*^ | 2 (3.4%) | **0.1 (0.1-0.3)** | 100.0 (2.5-100.0) | 61.1 (46.9-74.1) | N.D. | 0.078 (0.999) | 0.018 |
| Readmission | 4 (6.9%) | **4.8 (3.3-5.7)** | 25.0 (0.6-80.6) | 70.4 (56.4-82.0) | 0.57 (0.43-0.70; 0.514) | 6.142 (0.631) | 0.065 |
| Return to OR | 2 (3.4%) | 3.1 (2.7-4.2) | 50.0 (1.3-98.7) | 41.1 (28.1-55.0) | 0.51 (0.37-0.64; 0.976) | 8.280 (0.309) | 0.033 |
| Death | 1 (1.7%) | **0.2 (0.1-1.4)** | 100.0 (2.5-100.0) | 52.6 (39.0-66.0) | N.D. | 0.000 (N.D.) | 0.015 |
| Discharge to nursing/rehab facility | 28 (48.3%) | **8.4 (3.9-28.5)** | 75.0 (55.1-89.3) | 56.7 (37.4-74.5) | 0.72 (0.58-0.83; **0.002**) | 10.760 (0.149) | 0.323 |
| Sepsis | 1 (1.7%) | 1.0 (0.7-2.4) | 100.0 (2.5-100.0) | 43.9 (30.7-57.6) | 0.50 (0.34-0.66; 1.000) | 4.960 (0.549) | 0.017 |
| ***Pelvic ring/acetabular fracture (n=116)*** | |  |  |  |  |  |  |
| Serious complication | 23 (19.8%) | **6.1 (4.3-9.4)** | 30.4 (13.2-52.9) | 76.3 (66.4-84.5) | 0.64 (0.55-0.73; **0.021**) | 12.149 (0.145) | 0.171 |
| Any complication | 81 (69.8%) | **6.5 (4.5-9.5)** | 30.9 (21.1-42.1) | 97.1 (85.1-99.9) | 0.66 (0.57-0.75; **0.002**) | 9.637 (0.291) | 0.588 |
| Pneumonia | 12 (10.3%) | **0.3 (0.1-0.8)** | 66.7 (34.9-90.1) | 82.7 (74.0-89.4) | 0.77 (0.69-0.85; **0.001**) | 7.654 (0.176) | 0.100 |
| Cardiac complication | 1 (0.9%) | 0.2 (0.0-0.4) | 100.0 (2.5-100.0) | 75.7 (66.8-83.2) | N.D. | 0.066 (0.999) | 0.008 |
| Surgical site infection | 20 (17.2%) | **2.1 (0.9-2.8)** | 20.0 (5.7-43.7) | 83.3 (74.4-90.2) | 0.57 (0.48-0.66; 0.289) | 4.913 (0.767) | 0.165 |
| Urinary tract infection | 9 (7.8%) | **0.6 (0.4-1.1)** | 22.2 (2.8-60.0) | 82.2 (73.7-89.0) | 0.58 (0.49-0.67; 0.506) | 7.925 (0.339) | 0.076 |
| Venous Thromboembolism | 9 (7.8%) | **0.9 (0.6-1.3)** | 55.6 (21.2-86.3) | 80.4 (71.6-87.4) | 0.83 (0.75-0.89; **<0.001**) | 11.168 (0.192) | 0.075 |
| Renal failure ^*^ | 1 (0.9%) | 0.1 (0.0-0.2) | 100.0 (2.5-100.0) | 82.1 (73.8-88.7) | N.D. | 0.000 (1.000) | 0.008 |
| Readmission | 6 (5.2%) | **2.7 (1.9-3.9)** | 16.7 (0.4-64.1) | 90.9 (83.9-95.6) | 0.69 (0.60-0.78; **0.008**) | 9.279 (0.233) | 0.049 |
| Return to OR | 19 (16.4%) | **2.6 (1.5-3.5)** | 47.4 (24.4-71.1) | 67.0 (56.7-76.2) | 0.65 (0.56-0.74; **0.035**) | 8.204 (0.414) | 0.154 |
| Death | 1 (0.9%) | 0.1 (0.0-0.4) | 100.0 (2.5-100.0) | 77.4 (68.7-84.7) | N.D. | 0.142 (0.998) | 0.008 |
| Discharge to nursing/rehab facility | 55 (47.4%) | **21.2 (10.7-42.4)** | 34.5 (22.2-48.6) | 88.5 (77.8-95.3) | 0.63 (0.53-0.71; **0.021**) | 17.310 (**0.027**) | 0.279 |
| Sepsis | 2 (1.7%) | **0.3 (0.2-0.7)** | 100.0 (15.8-100.0) | 70.2 (60.9-78.4) | 0.85 (0.77-0.91; **0.005**) | 6.351 (0.385) | 0.017 |
| **Outcome** | **Observed**  **rate** | **Predicted**  **risk** | **Sensitivity**  **(95% CI)** | **Specificity**  **(95% CI)** | **AUC**  **(95% CI; p-value)** | **HL-test** | **Brier score** |
| ***Femoral fracture (n=261)*** |  |  |  |  |  |  |  |
| Serious complication | 29 (11.1%) | 8.7 (6.0-13.1) | 37.9 (20.7-57.7) | 69.4 (63.0-75.3) | 0.65 (0.59-0.71; **0.003**) | 5.783 (0.672) | 0.096 |
| Any complication | 150 (57.5%) | **8.4 (5.8-13.3)** | 40.0 (32.1-48.3) | 82.9 (74.6-89.4) | 0.69 (0.63-0.74; **<0.001**) | 6.012 (0.646) | 0.456 |
| Pneumonia | 16 (6.1%) | **1.2 (0.6-2.9)** | 68.8 (41.3-89.0) | 72.2 (66.2-77.8) | 0.73 (0.68-0.79; **0.001**) | 7.622 (0.471) | 0.058 |
| Cardiac complication | 4 (1.5%) | 0.8 (0.2-2.0) | 75.0 (19.4-99.4) | 73.9 (68.1-79.2) | 0.87 (0.83-0.91; **<0.001**) | 4.899 (0.672) | 0.014 |
| Surgical site infection | 15 (5.7%) | **0.9 (0.7-1.4)** | 40.0 (16.3-67.7) | 68.3 (62.1-74.1) | 0.53 (0.47-0.60; 0.723) | 15.583 (**0.049**) | 0.056 |
| Urinary tract infection | 17 (6.5%) | **2.1 (1.1-3.1)** | 41.2 (18.4-67.1) | 81.1 (75.7-85.9) | 0.69 (0.63-0.74; **0.002**) | 8.415 (0.394) | 0.062 |
| Venous Thromboembolism | 4 (1.5%) | 1.1 (0.8-1.8) | 0.0 (0.0-60.2) | 69.3 (63.2-74.8) | 0.54 (0.47-0.60; 0.729) | 5.849 (0.664) | 0.015 |
| Renal failure ^*^ | 9 (3.4%) | **0.2 (0.1-0.5)** | 50.0 (15.7-84.3) | 75.2 (69.3-80.5) | 0.77 (0.71-0.82; **0.001**) | 6.475 (0.372) | 0.031 |
| Readmission | 9 (3.4%) | 4.6 (2.7-7.7) | 66.7 (29.9-92.5) | 72.2 (66.3-77.7) | 0.75 (0.69-0.80; **0.005**) | 6.518 (0.589) | 0.033 |
| Return to OR | 16 (6.1%) | **1.7 (1.2-2.1)** | 50.0 (24.7-75.3) | 61.6 (55.2-67.8) | 0.56 (0.50-0.62; 0.393) | 3.305 (0.855) | 0.059 |
| Death | 13 (5.0%) | **0.8 (0.2-3.6)** | 76.9 (46.2-95.0) | 70.2 (64.0-75.8) | 0.83 (0.78-0.88; **<0.001**) | 11.285 (0.127) | 0.043 |
| Discharge to nursing/rehab facility | 125 (47.9%) | 47.7 (22.6-69.6) | 2.4 (0.5-6.9) | 97.1 (92.6-99.2) | 0.68 (0.62-0.74; **<0.001**) | 5.815 (0.668) | 0.233 |
| Sepsis | 3 (1.1%) | 1.0 (0.5-1.5) | 66.7 (9.4-99.2) | 53.5 (47.2-59.7) | 0.79 (0.74-0.84; **<0.001**) | 12.569 (0.128) | 0.011 |

Observed rate is shown as the number and percentage of patients with the given outcome in the study population, predicted risk is shown as the median (P_25_-P_75_) ACS NSQIP SRC-predicted risk of all patients with the given outcome. Sensitivity and specificity are given with 95% CI. The AUC is given with 95% CI and p-value. For the Hosmer-Lemeshow test, the Chi-squared value and associated p-value are given.

^*^Predicted risk is not computed for patients with acute renal failure or for patients on dialysis (3 for rib fractures, 3 for pelvic ring/acetabulum fracture, and 7 for femoral fracture).

Underestimated predicted complication rates are highlighted as bold.

AUC, area under the Receiver Operating Characteristic curve; HL, Hosmer-Lemeshow test; N.D., not determined; OR, operating room.
